# The effects of an enhanced simulation programme on medical students’ confidence responding to clinical deterioration

**DOI:** 10.1186/s12909-016-0685-2

**Published:** 2016-06-10

**Authors:** George Hogg, David Miller

**Affiliations:** School of Medicine, University of Dundee, Dundee, Scotland, UK; School of Education and Social Work, University of Dundee, Dundee, Scotland, UK; Clinical Skills Centre, University of Dundee School of Medicine, Ninewells Hospital and Medical School, Dundee, DD1 9SY UK

**Keywords:** Patient simulation, Simulation training, Failure to rescue, Critical illness

## Abstract

**Background:**

Clinical deterioration in adult hospital patients is an identified issue in healthcare practice globally. Teaching medical students to recognise and respond to the deteriorating patient is crucial if we are to address the issue in an effective way. The aim of this study was to evaluate the effects of an enhanced simulation exercise known as RADAR (Recognising Acute Deterioration: Active Response), on medical students’ confidence.

**Methods:**

A questionnaire survey was conducted; the instrument contained three sections. Section 1 focused on students’ perceptions of the learning experience; section 2 investigated confidence. Both sections employed Likert-type scales. A third section invited open responses. Questionnaires were distributed to a cohort of third-year medical students (*n* = 158) in the North East of Scotland 130 (82 %) were returned for analysis, employing IBM SPSS v18 and ANOVA techniques.

**Results:**

Students’ responses pointed to many benefits of the sessions. In the first section, students responded positively to the educational underpinning of the sessions, with all scores above 4.00 on a 5-point scale. There were clear learning outcomes; the sessions were active and engaging for students with an appropriate level of challenge and stress; they helped to integrate theory and practice; and effective feedback on their performance allowed students to reflect and learn from the experience. In section 2, the key finding was that scores for students’ confidence to recognise deterioration increased significantly (*p*. < .001) as a result of the sessions. Effect sizes (Eta^2^) were high, (0.68–0.75). In the open-ended questions, students pointed to many benefits of the RADAR course, including the opportunity to employ learned procedures in realistic scenarios.

**Conclusions:**

The use of this enhanced form of simulation with simulated patients and the judicious use of moulage is an effective method of increasing realism for medical students. Importantly, it gives them greater confidence in recognising and responding to clinical deterioration in adult patients. We recommend the use of RADAR as a safe and cost-effective approach in the area of clinical deterioration and suggest that there is a need to investigate its use with different patient groups.

## Background

Within the acute hospital setting many patients who require intensive care or have a cardiac arrest will have had clear and detectable signs of clinical deterioration during the hours preceding the adverse event [1–7]. If not recognised and managed promptly deterioration can lead to increased morbidity, protracted hospital stays and in some cases patient death [8, 9]_._ Until quite recently one of the major challenges facing clinical staff and medical educators was the lack of consensus as to what actually constitutes clinical deterioration. A definition by Jones et al [10] states that ‘*A deteriorating patient is one who moves from one clinical state to a worse clinical state which increases their individual risk of morbidity, including organ dysfunction, protracted hospital stay, disability or death’* (p 1031). Prior to this clear definition many studies referred to clinical deterioration in terms of the outcome in terms of infection and hospital mortality [11–13], Iatrogenesis and medical neglect [14–17], discrete clinical complications [6, 18, 19] and abnormal vital signs [20, 21]. Much of the work led to the development of national patient safety systems in the United States [22], Australia [23] and the United Kingdom [24]. The focus of this work included the detection of, and response to clinical deterioration using track and trigger systems with early warning scoring (EWS) systems and rapid response teams [25–28].

A systematic literature review [29] investigating the undergraduate training for medical students in the care of the acutely ill patient identified a consistent theme of lack of confidence and competence in the recognition and management of the acutely ill (deteriorating) adult. The same literature review [29] identified that junior doctors were sometimes still not confident to perform some acute care skills up to 3 years post qualification. This is a key challenge for medical education which is moving apace from the traditional didactic, teacher-centred model to a more integrated, interprofessional learning experience for medical students [30]. This shift is necessary to bridge the gap between the need to have a comprehensive medical education and preparation for the patient focussed realities of the clinical setting. It is therefore incumbent upon medical educators to ensure that teaching and programmes of learning are designed to prepare students effectively for their clinical role. In line with this ethos, the “Recognising Acute Deterioration: Active Response” (RADAR) course was developed as an innovative approach to using enhanced simulation to achieve the aims of improving undergraduate medical students’ confidence in this critical area of patient care.

The use of simulation in medical education is not new, having been used in resuscitation training since the 1960s. It ranges from the simple part-task trainer used to teach venepuncture (a rubber arm), through high fidelity human patient simulators (HPS) which electronically replicate physiology, to what Kneebone et al [31] describe as patient focused simulation using real people. While part-task trainers and HPS have benefits, for example the ability to allow students to practise invasive procedural skills e.g. cannulation, there are also important limitations. These include the inability of an HPS to reproduce fear, anxiety, restlessness, changes in physical appearance and conscious level. In order to overcome these limitations it was decided to recruit human volunteers to act as quasi patients for the RADAR course. These simulated patients were provided with a script to follow and were carefully prepared with theatre make-up to create an appropriate moulage [31]. Further details of the recruitment, preparation and briefing of these patients will be provided below.

This paper will briefly outline the structure of the RADAR programme using high-fidelity simulated patients, and focus on the evaluation related to its first presentation. Specifically we report evidence in relation to 1) students’ views of the nature of the learning experience; and 2) the effects of the learning experience on their confidence to recognise and respond to clinical deterioration. The aim of this study was to evaluate the effects of an enhanced simulation exercise known as RADAR (Recognising Acute Deterioration: Active Response), on medical students’ confidence.

## Methods

### Design and setting of the study

The study was one element of a longer-term action-research project.^1^ A questionnaire-based survey was conducted to investigate student perceptions of a new element of the medical education course: the RADAR programme. The questionnaire was anonymous, requesting no personal details, and consisted of three sections. The first two sections employed Likert-scale statements; the third contained four open-ended questions. The first section related to student perceptions of the educational content of the programme. For example, an item asked respondents to rate the new sessions in terms of how they ‘helped them to link theory and practice’. Responses were on a five-point scale, with higher scores representing more positive views. The second set of statements were designed to measure the impact of the RADAR teaching on students’ confidence to recognise and respond to clinical deterioration. For example, one item asked respondents to rate their confidence in terms of ‘Effective Communication during an acute episode’. Again, responses were on a five-point scale, with higher scores representing greater confidence. This second section of the scale was administered on three occasions; before teaching, after the morning Acute Medical Unit Ward Simulation Exercise (AMUWSE) sessions, and then after the RADAR sessions. Finally the third section of the instrument asked for additional comments to be added at the end of the teaching session. These were open-ended questions where respondents were encouraged to identify the most useful or interesting aspects of the course and invited to add any further comments.

The quantitative data from sections 1 and 2 were entered into IBM SPSS v18 and analysed. Repeated-measures analysis of variance techniques (ANOVA) were employed to investigate changes in scores following the learning experience, with post-hoc tests (Tukey HSD test) employed as appropriate. The Kolmogorov-Smirnov statistic was used to assess normality.

In order to minimise threats to the face validity and content validity of the questionnaire, the wording of the items directly related to the shared intentions of the RADAR sessions (section 1) and the processes that students had to employ during the session (section 2). In terms of reliability, Cronbach’s alpha figures were as follows: for the first section of the instrument, 0.98, representing excellent internal consistency; for the three administrations of section 2 of the instrument, figures were 0.80, 0.79 and 0.76 respectively, indicating good or acceptable levels of internal consistency.

The qualitative data from the open-ended questions in section three were examined through a simple content analysis. In terms of inter-rater reliability, data were examined independently by three data coders, with discrepancies being resolved through discussion. Analyses and interpretations were subsequently examined by two other researchers with backgrounds in education and medical education respectively.

The setting for the study was a University medical school in the North East of Scotland. The course followed is of 5 years duration and based on the outcomes and standards for undergraduate medical education stated in the General Medical Council publication ‘Tomorrow’s Doctors’ [32]. The Medical School involved uses a spiral curriculum based on a body systems approach throughout the first 3 years, followed by 2 years of clinically focused teaching.

### Participants

The AMUWSE and RADAR sessions are completed by students towards the end of year 3 during what is known as the transition block between theory (years 1–3) and practice (years 4 and 5). A purposive sampling approach was adopted; the sample for this study comprised all year three undergraduate medical students (*N* = 165) who were registered to attend the acute medicine teaching sessions. During the period of data collection 158 (95.7 %) of these students attended the sessions and of these 130 (82.2 %) completed questionnaires. Data collection took place during May and June 2014.

### The context

The RADAR programme was introduced as an addition to the students’ acute care teaching. In recent years, a key element of this teaching has been an acute medical ward simulation exercise (AMUWSE). The aim of the AMUWSE is to introduce medical students to the concept of acute medicine. It involves students working in pairs to manage simulated patients being admitted to an AMU. The aim is to introduce students to the differences in admitting patients with an acute illness, as opposed to the usual ‘routine’ cases which they have been involved with up to this point in their training. The AMUWSE approach effectively starts students thinking about acute illness and the concept of clinical deterioration, which is then specifically addressed in the subsequent RADAR sessions. The AMUWSE started as an inter-professional exercise for medical and nursing students [33] Since then it has continued to develop and be adapted to accommodate an undergraduate assessment [34], a postgraduate assessment for doctors in difficulty [35] and a teaching tool for newly qualified nurses [36]. The RADAR session has been designed to follow the AMUWSE exercise, and is conducted on the same day, in the afternoon session.

### Design and development of RADAR

The aim of RADAR is to introduce medical students to the concept of the recognition, response to, and rescue of, the deteriorating adult hospital patient in order to increase their confidence in managing such patients in real-life situations. Sessions are two hours long with an initial introductory presentation (15 min) in which the concept and challenges of clinical deterioration are discussed, the format of the rest of the session and student questions are addressed. This is followed by a briefing (10 min) in which the students are given direction on what is expected of them during the scenarios. A fundamental principle is that the students are expected to act as themselves and not role-play other people. This is seen as crucial as they are being prepared for the real world of clinical practice in which they may well be asked to see patients and discover that the patient is acutely unwell or deteriorating. It is therefore vital that the student is safe and can carry out an effective and appropriate response before seeking early senior help and escalation of care for the patient. The students are instructed to carry out an ABCDE assessment (Airway, Breathing, Circulation, Disability, and Examination), calling for help whenever they encounter a problem they cannot manage and to then handover to their senior using the SBAR (Situation, Background, Assessment, Recommendation) communication tool. Once the students have escalated care and submitted the handover they are expected to record what has happened during the patient scenario. The simulated scenarios are then 30 min long (10 min for the students to assess the patient and seek help and 20 min for debrief and feedback). By way of illustration, in one scenario the students receive a handover on a patient who has been admitted with shortness of breath, fever and confusion. The students are expected to assess the patency of the patient’s airway, count and record the respiratory rate, pulse oximetry and breath sounds. The tutor present will give the students the physiological parameters once the student has carried out the task. The students are expected to identify from the information given that the patient has a breathing problem which they are not capable of managing and to instigate the escalation protocol and ask for a registered nurse to review the patient. The tutor then assumes this role and takes a handover from the students. The scenario then stops and the feedback begins. The students work in small groups and see 3 different simulated patients.

### Recruitment and preparation of patients

The recruitment and preparation of the patients are of central importance to the functioning of the RADAR sessions. The patients are recruited from the bank of 180 volunteers available within the Clinical Skills Centre in which the study was based. They receive payment for travel expenses and parking only. They are trained by a full-time nurse trainer who is also responsible for the application of the make-up. The patients are all experienced and had completed the in-house training which includes an induction day, sessions on maintaining a character, portraying symptoms, moulage and communication. These sessions are facilitated by the nurse trainer employed by the centre. Before the RADAR sessions patients are prepared using short scripts which identify the main condition they are going to portray, specific instructions on the symptoms they should discuss and any other pertinent details on past medical history, current health status etc. On the day of the RADAR sessions the make-up is applied and any props are given to the patient by the trainer who also answers any questions the patient might have. For example in the hypovolaemic shock scenario the patient holds a small container of ice so that when the student introduces themselves the patient’s hand feel cold and clammy. They have make-up applied that makes them look pale and they are in a hospital bed looking tired.

The patients are expected to follow the script to ensure that each of the groups of students receive the same scenario, although small deviations are acceptable as RADAR is not an assessment which requires strict adherence and standardisation. Each group of students completes three scenarios. The scenario timings are specifically designed to be short (10 min) of patient interaction in order to reduce fatigue on the patient. The patient is allowed to come out of role and relax during the 20 min feedback session between tutor and students. The main difficulty associated with SPs is the number required to cover the sessions. In order to give all students the opportunity it requires 6 SPs per day over four days, and this highlights the value of a large pool of volunteers.

### The learning outcomes for the RADAR sessions

Students are expected to:

1. Discuss the use of the ABCDE approach to an acutely ill/deteriorating adult patient.
2. Discuss the differences in applying the ABCDE approach to a (simulated) patient who is unwell and a manikin requiring ‘resuscitation techniques’.
3. Demonstrate how to recognise a patient is unwell/deteriorating using the ABCDE approach with a simulated patient.
4. Identify that the simulated patient has changes in physiological parameters and calculate Scottish Early Warning Score (SEWS) score.
5. Interpret evidence from ABCDE and SEWS in collaboration with qualified clinician to develop an escalation of care plan.
6. Assemble evidence from the ABCDE and SEWS assessments and relay information to a qualified clinician using SBAR.
7. Summarise the recognition, recording, response and rescue of the patient during the scenario.

### Ethical considerations

These were applied throughout the research with university-level ethics committee approval granted prior to the start of the project (University of Dundee, UREC 10033). Student confidentiality and anonymity was guaranteed as was the reassurance that non-participation would not prejudice those students. Students were provided with an information sheet detailing the study, their level of engagement and the contact details of the lead investigator. Those who were willing to participate were asked to sign a consent form for the use of the anonymised data in subsequent publications.

## Results

Completed questionnaires were received from 130 students, 55 males and 75 females, with a mean age of 22 years. This was a response rate of 82 %. Section 1 of the questionnaire was based on 10 statements relating to the learning outcomes, student engagement with the sessions, feedback and general overview of the sessions from the students’ perspective. Students were asked to rate each of the statements on a Likert scale of 1 (Not at all) to 5 (To a large extent). Mean item scores and standard deviations were calculated and are presented in Table 1.

**Table 1:** Means and standard deviations for items in section 1 of the Student Questionnaire (*n* = 130) University of Dundee

The RADAR Sessions:	Mean Score (1–5)	Standard Deviation
Had clear learning outcomes	4.36	0.73
Kept me actively involved	4.68	0.59
Were relevant to my learning needs	4.67	0.58
Were appropriate for my level of experience	4.60	0.64
Were challenging without being threatening	4.58	0.71
Helped me to integrate theory and practice	4.64	0.59
Stimulated my interest	4.70	0.56
Encouraged me to think through a clinical problem myself	4.60	0.64
Provided me with effective feedback	4.44	0.64
Increased my readiness to use what I have learned in the clinical setting	4.46	0.71

It can be seen that the mean scores for each statement were all greater than 4, which indicates that the students were positive about the educational content of the sessions.

Section 2 of the questionnaire was designed to investigate students’ confidence in relation to specific aspects of the sessions and was based on seven statements using a Likert Scale of 1 (No Knowledge) to 5 (Greater Knowledge). This section of the instrument was administered on three occasions – first thing in the morning, after the morning session, and at the end of the day – in order to explore changes in students’ confidence in key areas as a result of the RADAR intervention.

A one-way repeated measures ANOVA was conducted to compare scores on the medical students’ confidence in knowledge at Time 1, Time 2 and Time 3 with the means and standard deviations presented in Table 2. There was a significant effect for time, Wilk’s lambda = 0.31, F (2,128) = 142.03, p < 0.001, partial eta squared = 0.68. That is, students gained significantly in confidence during the course of the sessions. Post-hoc comparisons were then conducted; these demonstrated that the gains between all time periods were significant at the .05 level. That is, students’ responses reflected greater confidence in key processes after the morning session, and they became more confident again after the afternoon session. The partial eta^2^ figure of 0.68 reflects a large effect size.

**Table 2:** Students’ overall confidence in ability to identify and manage immediately life-threatening emergencies (*n* = 130) University of Dundee

Time Period	N	Mean item score	Standard Deviation
Time 1 (Pre AMUWSE)	130	3.38	0.07
Time 2 (Post AMUWSE)	130	3.60	0.85
Time 3 (Post RADAR)	130	4.60	0.64

Finally, Table 3 shows the scores for each individual item in part 2 of the scale.

**Table 3:** Change in students’ confidence by item (*n* = 130) University of Dundee

Statement	Time 1		Time 2		Time 3			Effect size (Eta^2^)
	M	SD	M	SD	M	SD	*p*	
1. The ABCDE Approach	3.38	0.87	3.60	0.85	4.60	0.64	<.001	.68
2. What to do when I’m in over my head	2.58	1.13	3.22	1.04	4.22	0.75	<.001	.72
3. How to interpret observed rapid changes in the patient	2.78	0.94	3.31	0.87	4.29	0.66	<.001	.68
4. Effective communication during an acute episode	2.77	0.97	3.41	0.92	4.28	0.74	<.001	.71
5. Getting help from senior colleagues	2.81	1.12	3.44	1.08	4.40	0.77	<.001	.67
6. Approach to the specific emergencies covered	2.66	0.84	3.28	0.87	4.31	0.68	<.001	.75
7. Using SEWS and SBAR to call senior help	2.70	1.06	3.38	1.03	4.46	0.84	<.001	.68

This indicates improved confidence in each of the categories. It also shows high effect sizes in the eta^2^ column, pointing to the magnitude of these gains.

Section 3 of the questionnaire contained open-ended questions. The first asked for the top three things that students had learned. Of the 130 students, 108 provided responses. SBAR was the most frequently cited benefit from the sessions, with 78 nominations, followed by 77 for non-technical skills, 61 for ABCDE and 42 for communication. When asked about the most interesting aspects of the RADAR session, 125 of the 130 students offered comments, with a clear majority (79) noting the simulation. Of the other responses, non-technical skills received 37 nominations, with no other aspects receiving more than 20 (ABCDE). Secondly the students were asked for the least useful aspects of the session, or those that needed improvement. Of the 130 respondents, 60 stated ‘nothing’. Amongst a spread of other responses, 27 students suggested that the number of students in each group should be reduced (there were 10 students in each group). No other single factor reached double figures.

## Discussion

Based on the results of this study it can be seen that medical students’ confidence in relation to the clinical aspects of deterioration has increased as a direct result of the RADAR sessions. The findings indicate that the combination of situated learning within a realistic clinical simulation suite, working through different scenarios and feedback and debriefing can combine to enhance students’ confidence in recognising and responding to clinical deterioration. This is consistent with findings from previous studies using both simulators [37, 38] and simulated patients [39, 40] which support the use of simulation as an educational tool allowing students to practise procedures and make judgements without any harm or patient safety being compromised [41]. However, RADAR adds greater realism to the learning process. Rapid and realistic changes can be written into authentic scenarios and experienced in real time. This allows students to practise and make judgements on a crucial area of practice – the clinically deteriorating patient – in a safe and controlled learning environment.

From a teaching perspective, students were positive about the learning experiences provided. Amongst the features that appeared to contribute to the success were the learning outcomes, the sessions being challenging but not threatening, the integration of theory and practice, and the opportunity to think through problems. These point to RADAR being at a level commensurate with students’ knowledge and skills, and are important in the context of student confidence and future application of their learning in clinical practice.

Findings related to specific aspects of recognising and responding to clinical deterioration (ABCDE approach, teamwork, SEWS, SBAR, etc.) showed significant, positive changes in each case, with very high effect sizes. Technically speaking, effect size is a measure of the magnitude of the changes; however, it can also be viewed as the *educational* significance of the results. These figures would seem to indicate that from an educational perspective RADAR has been very effective. The open-ended responses were consistent with these findings, confirming the value to the students of the simulation as a context to employ the key processes involved. Taken together, the findings demonstrate that the scenarios – specifically their realism, relevance and appropriateness for the students’ knowledge – are highly valuable in terms of improving confidence in specific procedures and thus the overall confidence levels of the students.

In written comments added at the end of the questionnaires, many respondents stated that ABCDE was amongst the top three things that they had learned. Once students have carried out an initial assessment and responded to any life-threatening conditions using the ABCDE approach the next stage is to record their findings using the Scottish Early Warning Score (SEWS). SEWS is a tool used to detect early changes in a patients’ physiological parameters, indicating deterioration. SEWS is introduced to medical students early in year 1 and they practise using it throughout their time in clinical skills sessions. However, it would appear from the results of this study that being able to practise SEWS and SBAR in a realistic clinical setting with simulated patients increases students’ confidence in the use and combination of the tools to achieve escalation in care. The scores for ‘Using SEWS and SBAR to call senior help’ demonstrated clear gains. The increased realism that characterises RADAR would appear to enhance the quality of the contextual learning and improve medical students’ confidence in the use of SBAR significantly.

Realism is often perceived as crucial to encouraging engagement with simulation and it has been suggested that in order to achieve this educators must acknowledge that humans think about realism in (at least) three ways: physical, conceptual and emotional & experiential [42, 43] In terms of physical realism manikins are often seen as poor in terms of the body shape and size, ‘skin’ texture etc. Simulated patients can overcome these barriers to physical realism, which combined with moulage is critical in demonstrating to undergraduate students the changes in a patient’s physical appearance which occur during deterioration. Conceptual realism involves concepts such as diagnosis, decision making and thinking ahead, and is used in RADAR to encourage students to make the connection between changes in physical appearance and physiological changes. For example, if there is hypoxia the oxygen saturations will be decreased, or if there is significant blood loss, the blood pressure will drop. Finally emotional and experiential realism relates to the global experience of the scenario and how the student feels [44, 45]. We believe that this is where the real person (simulated patient) comes forward as they are able to portray fear and anxiety which challenges the students in a safe and controlled way in preparation for the real clinical setting. The careful combination of all three aspects of realism in RADAR allows students to engage, work together and link previous learning in order to enhance their preparation for practice.

In terms of the practicalities of using simulated patients, there are several issues that are worthy of note. These include the need to have effective moulage. Many readers will be aware of the use of moulage in advanced trauma courses where major injuries, haemorrhage, burns etc., are used to support learning. In RADAR the moulage is much more discrete and involves representing pallor, cyanosis of the fingertips, lips or ear lobes. Often glycerine is used to represent sweating, small containers of iced water held in the patient’s hand reproduces cold and clammy extremities and pear drop sweets give the classic smell of ketoacidosis on the patient’s breath. The authors are also fortunate in having access to a large patient bank with the services of a patient trainer, administrative support in terms of allocation to sessions and funding for patient travel expenses which others may not.

### Limitations

The current study focused on student experiences in one institution only and this inevitably raises issues of generalizability. While we acknowledge this as a potential limitation, we are unaware of reasons why the student demographic in this case should differ markedly from other similar institutions. As such, it seems reasonable to suggest that similar benefits might accrue to medical students elsewhere. However, there remains one important area for further investigation. In the introduction to this paper we cited studies that pointed to students’ lack of confidence and competence in the recognition and management of the acutely ill. The current study has only investigated confidence. While it is not unreasonable to suggest that confidence and competence often go hand-in-hand, clearly the relationship is more complex than this. There is a need now to investigate the extent to which increased confidence is reflected in subsequent levels of competence.

## Conclusions

Adult learning theory advocates that adults learn best when they are active and engaged in learner-centred activities and RADAR is set within that paradigm. The key issue of feedback and debriefing after a scenario was commented on by students; this allows them to identify their learning and encourages reflection. A valuable aspect of RADAR is that the feedback and the process of reflection are likely to be enhanced because students are experiencing a scenario that is much closer to real life. In this context, situated learning theory posits that action is grounded in the concrete situation and that instruction must be done in complex, social environments. We believe that RADAR is unique in terms of the realism of the clinical simulation suite, simulated patients and moulage, which combine to provide a safe but complex, realistic learning experience. In terms of preparing students for real practice we believe that giving them the confidence to recognise the signs of deterioration early and ask for senior help is a major step in addressing the challenges of clinical deterioration. Work is currently progressing locally to introduce an adapted RADAR into the work plans of the NHS patient safety team which will see it run with postgraduate medical staff and qualified nursing teams as well as nursing students.

## Abbreviations

ABCDE, Airway, Breathing, Circulation, Disability, Examination; AMUWSE, Acute Medical Unit Ward Simulation Exercise; RADAR, Recognising Acute Deterioration: Active Response; SBAR, Situation, Background, Assessment, Recommendation; SEWS, Scottish Early Warning Score.

## Footnotes

1 The longer-term action-research project involves an iterative process of modifications to several aspects of the clinical skills elements of the students’ course, the subsequent evaluation of the changes and the incorporation of the findings into the following cycle. To date, elements have included the introduction of RADAR and its initial evaluation; modifications to the programme after the first year in the light of the findings, and evaluation of the changes; the inclusion of different groups of students (e.g. nursing students); evaluations of students’ perceptions via different methods (e.g questionnaires, focus groups); investigating perceptions of tutors on the courses; and changes to the location of RADAR within the curriculum. Further details can be obtained from the first author.
